# Intraabdominal hypertension and Abdominal Compartment Syndrome in patients with COVID-19: an integrative review

**DOI:** 10.1590/0100-6991e-20233539-en

**Published:** 2023-05-04

**Authors:** ANA CLARA FREITAS GALVÃO SOARES COSTA, OLIVAL CIRILO LUCENA DA FONSECA

**Affiliations:** 1 - Faculdade de Medicina Uninassau - Recife - PE - Brasil; 2 - Hospital Universitário Oswaldo Cruz - Recife - PE - Brasil

**Keywords:** COVID-19, SARS-CoV-2, Intra-Abdominal Hypertension, Intensive Care Units, COVID-19, Hipertensão Intra-Abdominal, Unidades de Terapia Intensiva, SARS-CoV-2

## Abstract

The first cases of COVID-19 were diagnosed in China, rapidly evolving with worldwide spread, turning into a pandemic. A percentage of these patients develop the severe form of the disease and progress to respiratory distress syndrome, requiring support in Intensive Care Units. Intra-abdominal Hypertension and Abdominal Compartment Syndrome are characterized by increased intra-abdominal pressure, and are subject to several predisposing factors, such as mechanical ventilation assistance, extracorporeal membrane oxygenation, elevated PEEP, intestinal obstructions, excessive fluid replacement, major burns and coagulopathies. Hence, for the management of patients with severe COVID-19, there are numerous risk factors for the development of intra-abdominal hypertension and abdominal compartment syndrome. Therefore, this study proposes to analyze the variables that directly interfere with the increase in intra-abdominal pressure in patients with COVID-19, as well as the changes in the organic systems caused, through an integrative literature review. .

## RESUMO

Os primeiros casos de COVID-19 foram diagnosticados na China, evoluindo rapidamente com uma disseminação a nível mundial, transformando-se em uma pandemia. Uma porcentagem desses pacientes desenvolve a forma grave da doença e evolui com Síndrome do Desconforto Respiratório, necessitando de suporte em Unidades de Terapia Intensiva. A Hipertensão Intra-abdominal e a Síndrome Compartimental Abdominal são caracterizadas pelo aumento da pressão intra-abdominal, e estão sujeitas a diversos fatores predisponentes, como assistência por ventilação mecânica, oxigenação por membrana extracorpórea, PEEP elevada, obstruções intestinais, reposição excessiva de fluidos, grandes queimados e coagulopatias. Com isso, para o manejo dos pacientes com COVID-19 grave, numerosos são os fatores de risco para o desenvolvimento da Hipertensão intraabdominal e da Síndrome Compartimental Abdominal. Por isso, esse estudo se propõe a analisar as variáveis que interferem diretamente no aumento da pressão intra-abdominal em pacientes com COVID-19, assim como as alterações nos sistemas orgânicos provocadas, por meio de uma revisão integrativa da literatura.

## INTRODUCTION

In December 2019, numerous cases of pneumonia emerged in Wuhan, China, which on January 7, 2020 were attributed to a new strain of the human coronavirus, SARS-CoV-2, responsible for causing the COVID-19 disease. In March 2020, the World Health Organization (WHO) declared a pandemic, recognizing outbreaks of COVID-19 in several countries around the world^1^. Patients who develop the severe form of the disease and need admission to Intensive Care Units (ICUs) for continuous monitoring are more predisposed to the occurrence of complications, such as Acute Renal Failure (ARF), Intra-abdominal Hypertension (IAH), and Abdominal Compartment Syndrome (ACS)^2^.

IAH is defined as repeated or sustained intra-abdominal pressure (IAP) ≥12mmHg, and ACS is defined as sustained IAP ≥20mmHg, plus organ dysfunction or multisystem alteration, and this pathological pressure increase is related to several risk factors, such as prone position, coagulopathy, gastroparesis, mechanical ventilation (MV), high positive end-expiratory pressure (PEEP), among others^3^. IAH and ACS lead to numerous physiological disorders in the body, affecting the central nervous, respiratory, cardiovascular, hepatic, renal, gastrointestinal, and vascular systems^4^. Some of the predisposing factors cited for increased IAP are found in the intensive care environment, which is why IAH in patients admitted to the ICU can reach 50%, being twice as prevalent in patients on MV^5^.

Furthermore, the lack of understanding of how to manage severe cases of the disease may have contributed to the development of IAH and ACS in patients with COVID-19 admitted to the ICU, since there is a moderate lack of knowledge of clinical signs and the need to monitor IAP based on in risk factors, in addition to the correct application of guidelines and treatment steps for IAH and ACS^6^ ^,^ ^7^.

According to the WHO, 15% of patients with COVID-19 progress to hospitalization and oxygen therapy, and 5% need ICU admission8. In view of this, there is a possible relationship between COVID-19 and the development of IAH and evolution to ACS, since the dysfunctions caused by the viral infection (e.g. coagulopathy and pneumonia) and the necessary management for these patients (MV, extracorporeal membrane oxygenation - ECMO -, prone positioning, volume resuscitation with fluids) are risk factors for increased IAP and the development of IAH and ACS^3^ ^,^ ^9^.

Through a literature review, this study aims to identify the variables directly related to COVID-19 and its management, with the development of Intra-abdominal Hypertension and Abdominal Compartment Syndrome, as well as the physiological impacts arising in such patients.

## METHODS

This study is an integrative qualitative review. The search was carried out in the PubMed database using the descriptors “abdominal compartment syndrome in COVID” and “intra-abdominal hypertension in COVID”, covering articles from 2019 to 2022. After excluding repeated works, we found 22 articles, to which we applied the eligibility criteria: works in English, with adult patients, which contained the descriptors in the abstract, and available in full text. We excluded literature reviews and articles outside the eligibility criteria. In the end, we selected five articles for inclusion in this review.

## RESULTS

Of the five articles, four are case reports and one is a retrospective cohort.

A relationship between IAP elevation and the development of ARF has been reported and should be considered in patients with worsening renal function and risk factors for the development of IAH and ACS^11^ ^,^ ^13^.

The anticoagulation performed in patients undergoing ECMO appears to be a contributing factor to the development of IAH and ACS, in addition to the circuit itself and the shearing stress of the device contributing for the development of a coagulopathy^2^.

There are also reports of positive fluid balance, fluid overload, and abdominal distention^11^ as risk factors in patients with COVID-19, in addition to an elevated PEEP^13^.

## DISCUSSION

According to the consensus and clinical practice guideline of the World Society of Abdominal Compartment Syndrome (WSACS)^3^, the normal IAP in healthy adults ranges from 0 to 5mmHg, and in critically ill patients it is tolerated when between 5 and 7mmHg. When this pressure pathologically rises to levels ≥12mmHg and is maintained, it is called IAH. Moreover, ACS is defined as IAP maintained above 20mmHg and associated with organ dysfunction or failure^3^ ^,^ ^7^ ^,^ ^14^. In patients with COVID-19, increased IAP is related to several risk factors intrinsic to organ dysfunctions caused by SARS-CoV-2 and to the therapeutic management of critically ill patients.

### Risk factors:

#### • COVID-19-Induced Coagulopathy and the Pro-Inflammatory State

The mechanisms of coagulation disorders caused by COVID-19 have been elucidated since the beginning of the pandemic. SARS-CoV-2 uses angiotensin-converting enzyme II to facilitate entry into cells. Through this mechanism, the infection begins, causing several cellular disturbances, impairing physiological functions. In healthy endothelial cells, there is synthesis of nitric oxide, an important mediator that inhibits the adhesion of leukocytes and platelets, prevents apoptosis, and counters inflammation, avoiding the migration of inflammatory cells to the vessel wall^15^. 

Infected endothelial cells have their nitric oxide production impaired, so that there is an increase in the patient’s inflammatory state and an imbalance in the regulation of endothelial coagulation mechanisms, leaving the patient with a condition of hypercoagulability. This disorder initially leads to the formation of thrombi in small vessels, impairing microcirculation and generating areas of ischemia. Furthermore, the pro-inflammatory state increases vascular permeability, causing interstitial edema and decreasing intravascular volume^15^ ^,^ ^16^.

The abdominal wall is extremely vascularized, as are the organs in this cavity, and with the state of hypercoagulability, ischemic areas, and reduced intraluminal volume, there is an increase in IAP as a compensatory mechanism to reestablish and maintain adequate organ perfusion. However, IAP elevation reduces venous return from the retroperitoneum and inferior vena cava, as well as hepatic artery and portal vein flows, further impairing capillary perfusion, which decreases inversely proportional to IAP elevation, creating a mechanism of feedback. Thus, with the gradual and sustained increase in IAP, IAH and then ACS become appropriate diagnoses, causing changes at the systemic level^15^ ^-^ ^18^.

#### • Mechanical ventilation

MV support is a known risk factor for the development of ACS, as well as high PEEP and the prone position (widely used in patients with COVID-19), since they transmit pressure through the diaphragm and act by reducing abdominal compliance^3^ ^,^ ^9^ ^,^ ^14^ ^,^ ^19^ ^,^ ^20^. Many critically ill patients admitted to the ICU require invasive ventilatory support, and by March 2020 in China, 3.2% of patients infected with SARS-CoV-2 evolved with intubation and MV^21^.

In MV, there is an expected variation in tidal volume and IAP, since this pressure in patients on spontaneous ventilation is 0mmHg and increases in MV due to the transmission of intrathoracic pressure through the diaphragm^19^. PEEP, on the other hand, influences perfusion and pressure parameters, since the greater the tension to prevent alveolar collapse, the greater the transpulmonary pressures and the lower the venous return^13^. In patients with COVID-19, PEEP can be titrated in different ways to optimize the ventilation/perfusion ratio in these patients, such as through the “PEEP table ARDSnet” or in “mini-titrations”^21^. In both cases, by raising this pressure, IAP also increases, and together with other aspects related to MV, high PEEP becomes an important predisposing factor for the development of IAH and ACS.

Prone positioning during MV is a strategy widely used in patients with Acute Respiratory Distress Syndrome, to optimize the ventilation/perfusion ratio and to recruit the dorsal lung areas that are more collapsed in the supine position. In patients with COVID-19, there is a recommendation to perform the maneuvers for the prone position in those who maintain an oxygen partial pressure/inspired oxygen fraction ratio <50mmHg^21^. This change in decubitus reduces abdominal compliance, which generates an increase in IAP, since the abdominal walls lose their ability to limit this pressure, leading to the development of IAH and ACS^17^ ^,^ ^19^.

#### • Extracorporeal membrane oxygenation (ECMO)

In patients with COVID-19 who have refractory hypoxemia and who may have reversible respiratory failure, the use of ECMO as an alveolar rescue mechanism is an alternative^21^. The relationship between ECMO and IAH and ACS involves inadequate venous return from the vena cava to the system, organ perfusion compromised by increased vascular resistance resulting from intra-abdominal pressure, and transmission of this pressure to the thorax, impairing lung mechanics and gas exchange^2^. It is thus a process that feeds back, since the failure of the pulmonary exchange mechanisms implies hypoxia and systemic suffering of the organs, causing the IAP to rise to maintain the oxygenation/perfusion ratio. In patients with ACS that are on ECMO, decompressive laparotomy can generate immediate benefit, but in those who are likely to need this support for a longer time, laparotomy can increase the risk of bleeding complications^2^.

### • Volume resuscitation:

Water overload and positive water balance in critically ill patients contribute to an increase in IAP and predispose to the development of IAH and ACS^3^ ^,^ ^5^ ^,^ ^14^ ^,^ ^20^. This occurs because there is capillary leakage, reducing the intravascular volume, causing the IAP to rise to maintain adequate tissue perfusion^5^ ^,^ ^18^ ^,^ ^19^.

#### Systemic repercussions:

The increase in IAP generates local and systemic hemodynamic repercussions by reducing arterial flow and venous return. One of the affected systems is the renal system, in which ACS generates a reduction in renal perfusion pressure and glomerular filtration rate^16^ ^-^ ^18^ ^,^ ^22^. The study carried out by Dupont et al.^13^ with a sample of 91 patients with COVID-19 admitted to the ICU showed that 17 patients on MV support developed severe ARF, with IAP levels >12mmHg. However, after decompression through laparotomy, glomerular filtration levels were quickly restored.

Furthermore, the presence of ACS significantly increases the mortality of critically ill patients admitted to the ICU^5^. In the studies analyzed in this review, some patients died, either directly due to the pathological increase in IAP or due to the systemic repercussions of IAH and ACS. Due to capillary leakage involved in the pathophysiology of IAH and ACS, bacterial translocation may occur, predisposing to complications such as sepsis and septic shock^23^, as described in the study by Kahana et al.^2^, in which the patient died of septic shock developed after the diagnosis of ACS. Also described by Alfaro et al.^11^ was a patient’s death after the decompressive laparotomy was not performed (even after medical indication), by option of the family members. In the latter case, the patient was treated with conservative measures, but they were not enough. In the retrospective study carried out by Dupont et al.^13^, there was a mortality of 72.7% of patients admitted with COVID-19 to the ICU who were diagnosed with ACS.

#### Diagnosis of IAH and ACS

IAP can be measured in several ways, but the one recommended by the WSACS is the transvesical, as it is less invasive and less costly. Theoretically, when there is any risk factor for the development of IAH and ACS in critically ill patients, IAP should be included in routine care as a protocol monitoring^3^. Some signs on the physical examination of patients may also indicate a possible increase in IAP, such as a tense and distended abdomen, jugular stasis, peripheral edema, and signs of acute pulmonary edema. An awaken patient may report abdominal pain, dyspnea, nausea, and vomiting^17^.

Cadan et al.^24^ described Doppler ultrasonography as a means of diagnosing IAH, associating the renal resistance index (RRI) with IAP. They prospectively measured the RRI in both kidneys, using the pathological value of this resistance, which is above 0.70, as a cutoff for the diagnosis of IAH^24^. Importantly, this is a non-invasive and widely accessible way of diagnosing IAH, which can be performed at the bedside. 

#### Treatment of IAH and ACS

The management of IAP elevation is based on steps that seek to nullify the disturbances caused in each of its pathophysiological pathways. It consists of evacuating intraluminal content, reducing extraluminal volume, reestablishing abdominal compliance, and optimizing water balance and tissue perfusion (Figure 2)^3^ ^,^ ^14^. The measures are initially conservative, to reduce the level of invasive intervention, since most patients are in severe conditions. However, in cases of ACS, in which the IAP is in grades III or IV and there is new organ dysfunction, decompressive laparotomy is the most indicated treatment when there is no therapeutic response after three consecutive serial measurements of the IAP, to quickly reduce local and systemic repercussions^3^ ^,^ ^16^ ^-^ ^19^. 

[Fig f1]

**Figure 1 f1:**
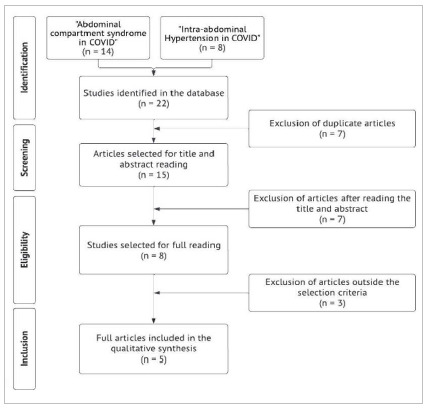
PRISMA flow diagram.

[Fig f2]

**Figure 2 f2:**
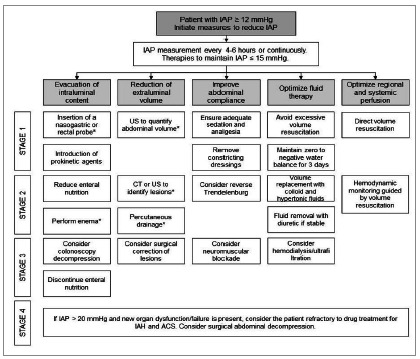
AIH and ACS treatment algorithm. CT: computed tomography; US: ultrasound; *Interventions in which the use of ultrasound can be an adjuvant tool. Adapted from Kirkpatrick AW, Roberts DJ, de Waele J, et al. Intra-abdominal hypertension and the abdominal compartment syndrome: updated consensus definitions and clinical practice guidelines from the World Society of the Abdominal Compartment Syndrome. Intensive Care Med 2013; 39: 1190-1206.

[Table t1]

**Table 1 t1:** Initial conditions of the patient.

Title	Age	Comorbidity	IAP monitoring	Volume resuscitation	MV
Decompressive Laparotomy for Veno-Venous Extracorporeal Membrane Oxygenation Failure due to Intra-Abdominal Hypertension in Critically Ill COVID-19 Patient^2^	53	NR	No	NR	Yes
Spontaneous liver rupture following SARS-CoV-2 infection in late pregnancy: A case report^9^	32	No	No	Yes	No
Chryseobacterium Indologenes Sepsis and Acute Renal Failure Secondary to Abdominal Compartment Syndrome in a Confirmed COVID-19 Patient^10^	70	Hypertension and obesity	No	Yes	Yes
COVID-19, Necrotizing Pancreatitis, and Abdominal Compartment Syndrome: A Perfect Cytokine Storm?^11^	37	Chronic pancreatitis and alcohol use disorder	No	Yes	Yes
Title	Age	Comorbidity	IAP monitoring	Volume resuscitation	MV
Severe Intraabdominal Hypertension in Critically Ill COVID-19 Patients With Acute Kidney Injury^12^	69*	Hypertension (76.5%), Diabetes (41.2%), high BMI**	Yes	Yes	Yes (70.3%)***

NR: not reported; *Median age; ** Percentages corresponding to patients with ARF in the cohort (n=17); ***Percentage referring to the number of patients who required MV within the study cohort (n=91).

[Table t2]

**Table 2 t2:** Patients’ evolution.

Title	ECMO	ARF	Decompressive Laparotomy	Death
Decompressive Laparotomy for Veno -Venous Extracorporeal Membrane Oxygenation Failure due to Intra-Abdominal Hypertension in Critically Ill COVID-19 Patient^2^	Yes	NR	Yes	Yes
Spontaneous liver rupture following SARS-CoV-2 infection in late pregnancy: A case report^10^	No	No	No	No
Chryseobacterium Indologenes Sepsis and Acute Renal Failure Secondary to Abdominal Compartment Syndrome in a Confirmed COVID-19 Patient^11^	No	Yes	No	Yes
COVID-19, Necrotizing Pancreatitis, and Abdominal Compartment Syndrome: A Perfect Cytokine Storm?^12^	No	Yes	No	No
Severe Intraabdominal Hypertension in Critically Ill COVID-19 Patients With Acute Kidney Injury^13^	NR	Yes	NR	77.2%*

NR: not reported; *Percentage referring to the sample of patients in the study who had COVID-19 and ACS.

Abdominal point of care ultrasound (POCUS) in the treatment of IAH and ACS has been described as an adjuvant tool in the stages recommended by the WSACS^7,25 27^. The first stage of treatment, as a measure of evacuation of intraluminal content, is the introduction of a nasogastric tube. The use of POCUS at this stage helps guide the introduction of the catheter, as well as confirmation of its positioning, with 100% accuracy, without subjecting the patient to radiation^7^ ^,^ ^25^ ^,^ ^26^.

POCUS is also used in the second stage of treatment, to assess the presence of free abdominal fluid, intestinal motility, pathological movements, the need for enema, and to assist in percutaneous drainage, when necessary^7^ ^,^ ^25^ ^,^ ^26^. In helping percutaneous drainage, ultrasound was comparable to abdominal radiography, but superior in determining gastric content, since it allows a better distinction between solid and liquid^25^.

#### Prevention

Prevention of IAP elevation primarily consists of controlling risk factors, since these are exceptionally prevalent in the intensive care setting^3^ ^,^ ^14^ ^,^ ^23^ ^,^ ^27^. Protocol monitoring of IAP is a rational choice in critically ill patients, especially those with COVID-19, as the dysfunctions caused by IAH and ACS can worsen the course of the disease and strongly alter mortality^2^ ^,^ ^13^.

## CONCLUSION

It is of paramount importance that the professionals of the multidisciplinary team involved in the care of patients with COVID-19 recognize the risk factors for the development of IAH and ACS, as well as the associated pathophysiology, to reduce the probability of IAP elevation. As a limitation, there is the small number of articles directly related to the topic, so more retrospective studies are needed for a detailed analysis.
